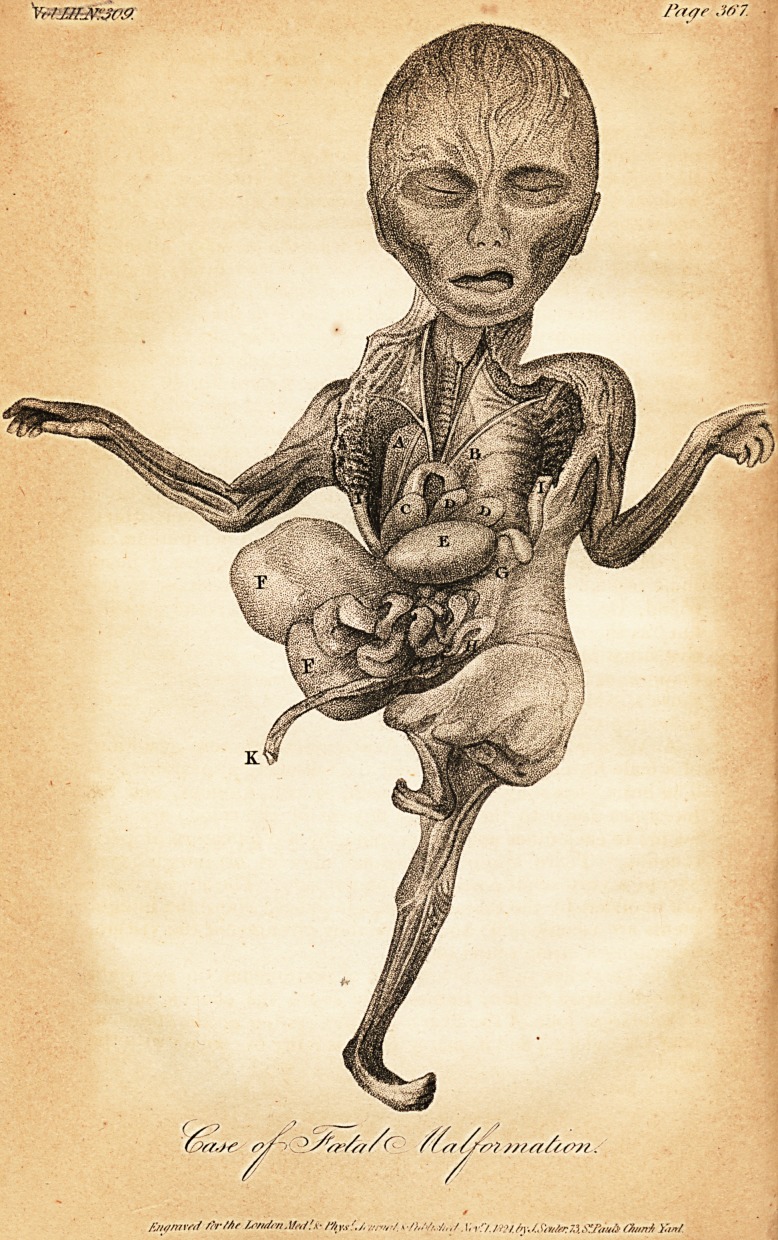# A Remarkable Instance of Fatal Malformation

**Published:** 1824-11

**Authors:** J. C. Yeatman


### ART. II.—*A remarkable Instance of fatal Malformation.* Communicated by J. C. YEATMAN, Esq.

[With an Engraving.]

MANY instances of fatal malformation are recorded in the fifty-one volumes of this truly valuable Journal. Among others are the following:—A fœtus with one eye in the forehead, another without a head; one without a brain, another without a bladder; one without a heart, another without arms or legs; one which had neither head nor heart, another with the placenta attached to the head; one with a tumor as large as the head, another defective in the brain and spine; one found in the body of a youth, another in the urinary bladder; one in the abdomen of a woman, eighty-three years of age,—another, in an ossified state, in a woman sixty years old; one in which all the viscera were double, another in which the viscera were upside down; one in which the viscera belonging to the right side of the thorax and abdomen were found in the left, and *vice versa;* and, lastly, not to multiply more instances of malformation, one born without abdominal muscles, the viscera being exposed to view. This last mentioned is described by Mr. Humby, in the third volume, page 137, in a communication dated St. Albans-street, January 15th, 1800; and it is stated to be in the museum of Mr. Heavyside. I allude to it in this place, because it bears some resemblance to the one now under the inspection of my friend, Dr. Edward Seagrim, (of Bratton, Wilts,) and myself; but, as there is a striking difference between them in the relative situation of several of the viscera, and in there being no diaphragm in the fœtus to be described, I beg leave to add it to those already recorded, leaving the causes of such phenomena to be explained by future physiologists.

A. W. miscarried, about the fourth month of utero-gestation, of a male fœtus, which presented the following appearances:—The heart, left lung, liver, stomach, spleen, kidneys, and the intestines down to the sigmoid flexure of the colon, are connected to each other and to the spine, by a duplicature of peritoneum. There are no abdominal muscles or integuments, except a very small portion on the left side. The above viscera are bounded by the thorax, loins, and pubes, where the integuments are rounded off, and where they circumvent the viscera, describing a circle round them.

The heart lies in an investment of peritoneum, in the right hypochondriac region, immediately above the convex surface of the larger lobe of the liver; its apex resting on the stomach, near the pylorus, and its margo obtusus lying in contact with the left lung.

The left lung, which is particularly small, consists of two lobes, lying in the epigastric region, over the lesser curvature of the stomach; its smaller lobe touching the heart.

The stomach occupies its usual situation. The spleen is connected to the stomach at its large extremity. There is no appearance of omentum.

The liver, consisting of two lobes, is unusually large, covering the stomach, spleen, kidneys, and most of the intestines; but in the Plate it is thrown aside, its concave part being uppermost, in order to bring the viscera into view. The convex surface of the larger lobe of the liver is bound down to the integuments, near the right hypochondriac region, by a broad and strong duplicature of cuticle.

The left kidney is situated below the spleen and the larger curvature of the stomach, deriving a cuticular covering from the integuments of the left side and loin, which is blended with, and lost in, the peritoneal coat of the intestines.

The right kidney is situated between the fœtal extremity of the funis, and the duplicature of cuticle which binds down the larger lobe of the liver.

On opening into the thorax, no diaphragm is perceivable.

The right lung, which consists of one lobe, occupies its usual situation, while nothing is contained in the left cavity of the thorax, and that cavity is much narrowed by a lateral incurvation of the spine.

The aorta, trachea, and œsophagus, lie on the right of the incurvated spine.

The distribution of the blood-vessels is natural, with the exception of the superior cava, which passes to the right auricle of the heart, over the right lung, throughout the full length of that organ.

The left extremity reaches half-way down the thigh of the right. The left thigh lies over the inguin, and is bound down by integument to the pubes. The heads of the tibia and fibula are connected to the inner condyle of the femur, passing from thence at a right angle; so likewise the astragalus, with respect to the internal maleolus.

A. W. states that, about a fortnight after conception, while walking through a lane, a cow broke through a hedge into the road, close to her; which so frightened her, that she ran a short distance, and jumped into a dry ditch, remaining there till the animal was out of sight.

My senior apprentice, Mr. Miles, who very properly treated the case of A. W., informs me, that the funis and placenta were natural.

It is my intention to deposit the fœtus in the museum of the Royal College of Surgeons.

*Description of the Plate.*

A. Right Lung, with the superior cava passing over it.
B. Incurvated Spine.
C. Heart.
D, D. Left Lung.
E. Stomach (inflated), and Spleen.
F, F. Liver.
G. Situation of the left Kidney, and the boundary of the integuments of the left side and loin.
H. Sigmoid flexure of the Colon.
I, I. Points at which the integuments terminated, superiorly and anteriorly, and which, previous to opening into the thoracic cavity, were joined together in the situation of the xyphoid cartilage, and immediately over the arch of the aorta.
K. Funis.

*Frome, Somerset; September 21st,* 1824.

√

## Figures and Tables

**Figure f1:**